# Superefficient optical frequency division referenced to μHz Schawlow-Townes-linewidth quantum noise–limited lasers

**DOI:** 10.1126/sciadv.aed1856

**Published:** 2026-06-17

**Authors:** Jiahao Hu, Yanlan Xiao, Honglei Yang, Siyi Xue, Wenchan Dong, Kunpeng Zhai, Sha Zhu, Kun Qiu, Shengkang Zhang, Jun Ge, Ning Hua Zhu, Xiaoshun Jiang, Jing Xu, Huashun Wen, Heng Zhou

**Affiliations:** ^1^ School of Information and Communication Engineering, University of Electronic Science and Technology of China, Chengdu 611731, China.; ^2^ Institute of Intelligent Photonics, Nankai University, Tianjin 300071, China.; ^3^ National Key Laboratory of Metrology and Calibration, Beijing Institute of Radio Metrology and Measurement, Beijing 100854, China.; ^4^ Wuhan National Laboratory for Optoelectronics, Huazhong University of Science and Technology, Wuhan 430074, China.; ^5^ National Laboratory of Solid State Microstructures, College of Engineering and Applied Sciences and School of Physics, Nanjing University, Nanjing 210093, China.

## Abstract

Optical frequency division (OFD) converts ultrastable optical frequencies to microwaves via an optical frequency comb, generating microwave oscillators with record-low phase noise and time jitter. However, conventional OFD systems face a notable trade-off between division complexity and noise suppression because of severe thermal and technical noise in optical references. Here, we address this challenge by using common-cavity bicolor Brillouin lasers as references, operating at the fundamental quantum noise limit with a 10-microhertz Schawlow-Townes linewidth. Enabled by these ultracoherent lasers, our OFD system uses a markedly simplified comb divider with an unprecedented division factor of 10, producing a 10-gigahertz microwave signal with exceptional phase noise of −65 decibels relative to the carrier per hertz at 1-hertz offset, −155 decibels relative to the carrier per hertz at 10-kilohertz offset, and −172 decibels relative to the carrier per hertz at 10-megahertz offset. Leveraging this purity, we implement broadband synthesis from 5 to 20 gigahertz with millisecond tuning. This work redefines the trade-off between noise suppression and division complexity in OFD, paving the way for compact, high-performance microwave synthesis for next-generation atomic clocks, quantum sensors, and low-noise radar systems.

## Teaser

Quantum noise–limited lasers enable simplified optical frequency division to generate microwaves with ultralow phase noise.

## INTRODUCTION

Ultralow-noise microwave signals are pivotal for precision measurement, radar systems, communication networks, timekeeping, and quantum technologies (*1*–*5*). Conventional electronic microwave sources are fundamentally limited by the Leeson effect, which dictates that phase noise of an electronic oscillator scales quadratically with its dissipation rate and carrier frequency (*6*, *7*). Optical frequency division (OFD) has emerged as a transformative solution to synthesize microwave signals with record-high phase coherence (*8*–*12*) by down-converting the superior stability of optical-frequency references (hundreds of terahertz) to microwave frequencies (gigahertz) using an optical frequency comb divider. The basic mechanism of OFD is to use two stable frequency references ($f_{\text{REF}1}$ and $f_{\text{REF}2}$) to lock the corresponding modes of the optical frequency comb, so the references and comb frequencies are related by the formula (*11*–*13*)

$$f_{\text{REF}1}-f_{\text{REF}2}=N\cdot f_{\text{spc}}+f_{\text{CEO}}+f_{\text{IF}}$$ (1)

Here, $f_{\text{spc}}$ denotes the frequency spacing between adjacent comb lines, which is also the output frequency of the OFD system. *N* is an integral, denoting the number of comb teeth between the two frequency references, and essentially, the OFD ratio. $f_{\text{CEO}}$ is the carrier-envelope offset frequency of the comb, and $f_{\text{IF}}$ denotes the intermediate frequencies (IFs) during frequency locking. Assuming that the comb divider and $f_{\text{IF}}$ introduce negligible phase noises (*14*), we can obtain (*12*, *15*)

$$S_{\text{OFD}}\left(f\right)=\left[∆S_{\text{REF}}\left(f\right)-S_{\text{CEO}}\left(f\right)\right]/N^{2}$$ (2)

In Eq. 2, $S_{\text{OFD}}$, $∆S_{\text{REF}}$, and $S_{\text{CEO}}$ denote the phase noise power spectral density of the OFD output frequency $f_{\text{spc}}$, the relative phase noise between the reference frequencies $f_{\text{REF}1}$ and $f_{\text{REF}2}$, and the comb’s carrier-envelope offset frequency $f_{\text{CEO}}$, respectively.

**Fig. 1.**
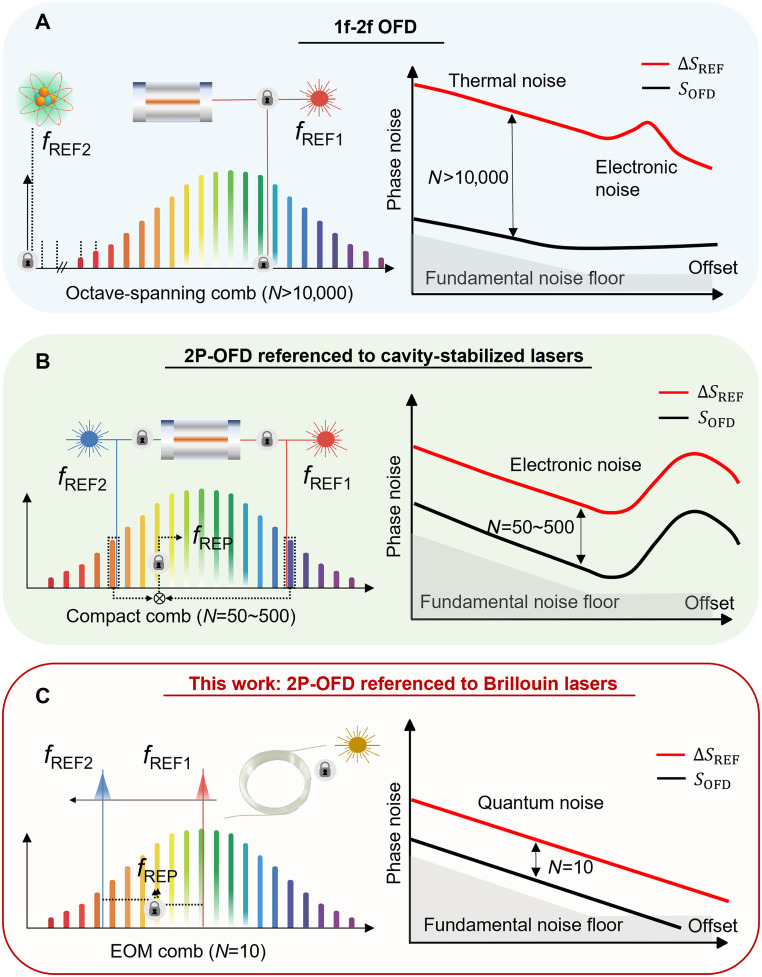
Comparison of OFD architectures. (**A**) Traditional architecture of a 1f-2f OFD system referenced to a cavity-stabilized laser (*f*_REF1_) and an atomic clock (*f*_REF2_), entailing an octave-spanning comb and large division ratio (*N* > 10,000) to coherently link the *f*_REF1_ and *f*_REF2_. (**B**) Architecture of a 2P-OFD system referenced to two cavity-stabilized lasers (*f*_REF1_ and *f*_REF2_). A compact comb can be used to implement modest division ratio (*N* = 50 to 500). However, 2P-OFD is prominently influenced by the electronic noise of the laser locking loops, and the inherent servo bump at a high offset frequency is hard to solve. (**C**) Architecture of our 2P-OFD system referenced to two-color Brillouin lasers (*f*_REF1_ and *f*_REF2_), which operate on the quantum noise limit with superior mutual coherence, allowing an unprecedentedly small division ratio (*N* = 10) to realize the top-level phase noise metrics of the OFD output signal.

It is clear from Eq. 2 that to generate a microwave signal with ultralow phase noise $S_{\text{OFD}}$, we can either reduce the relative phase noise between the two references $∆S_{\text{REF}}$ or increase the division ratio *N* (note that increasing *N* simultaneously increases the value of $f_{\text{REF}1}-f_{\text{REF}2}$). As shown in Fig. 1A, for traditional OFD based on 1f-2f interferometry (*11*, *12*, *16*–*22*), an octave-spanning optical frequency comb is adopted; one of its comb mode is locked to an optical reference $f_{\text{REF}1}$, which is commonly derived from an ultra-table laser Pound-Drever-Hall (PDH) locked to a high-finesse vacuum-enclosed Fabry-Pérot (FP) cavity; and the comb’s $f_{\text{CEO}}$ is locked to a radio-frequency (RF) signal derived from an atomic clock (e.g., rubidium or hydrogen maser). Given that $f_{\text{REF}1}$ is more than four orders of magnitude larger than $f_{\text{CEO}}$, this configuration achieves division ratio *N* > 10^4^, enabling substantial suppression of the phase noise $∆S_{\text{REF}}$ that is dominated by the thermal optical noise of the FP cavity (see Fig. 1A) (*11*, *12*). Despite its success in generating the purest microwave signals to date (*12*), 1f-2f OFD relies on a multitude of complex optical modules and electronic circuits, making the system prohibitively expensive and bulky, environmentally sensitive, and confined to laboratory settings.

An alternative method is two-point OFD (2P-OFD) (*23*–*33*). As shown in Fig. 1B, 2P-OFD uses two optical references (i.e., both $f_{\text{REF}1}$ and $f_{\text{REF}2}$ are optical), typically obtained by locking two lasers toward two different resonances of the same reference cavity, and these two lasers are used to stabilize the repetition frequency of a frequency comb. In 2P-OFD, the performance of the system does not depend on the carrier-envelope offset frequency $f_{\text{CEO}}$; thus, Eq. 1 can be represented as Eq. 3

fREF1−fREF2=Nfspc+fIF (3)

In addition, Eq. 2 can be represented as $S_{\text{OFD}}\left(f\right)=∆S_{\text{REF}}\left(f\right)/N^{2}$. The key merit of 2P-OFD is the common-mode noise rejection (CMNR) of the cavity thermal noise between $f_{\text{REF}1}$ and $f_{\text{REF}2}$, which substantially reduces the noise level of $∆S_{\text{REF}}$ compared to the 1f-2f interferometry. Consequently, minimized $∆S_{\text{REF}}$ relaxes the required division factor *N* and allows simplified and compact comb divider architectures, such as Kerr microcombs (*23*, *26*, *28*, *29*, *32*, *33*) and electro-optic modulation (EOM) combs (*24*, *25*, *27*, *30*, *31*). However, while thermal noise is suppressed, electronic noise in the laser locking system emerge as the dominant limitation of 2P-OFD (*31*, *34*, *35*). In particular, residual amplitude modulation noise (*36*, *37*), uncorrelated light-path fluctuations (*38*), and servo bumps of the laser locking loops (*23*, *29*–*33*) add excessive electronic noise to $∆S_{\text{REF}}$, preventing access to the intrinsic stability of the optical references.

Here, we demonstrate a type 2P-OFD leveraging common-cavity two-color Brillouin lasers as the optical references (see Fig. 1C), which operate at the fundamental quantum noise limit with Schawlow-Townes (ST) linewidth on the 10 microhertz level (*39*, *40*). Thermal noise and electronic noise are thoroughly suppressed via common-cavity noise rejection and coherently pumped bichromatic Brillouin lasers, achieving superb coherence between the reference lasers (i.e., ultralow phase noise $∆S_{\text{REF}}$) to overcome the substantial trade-off between division complexity and noise suppression. On the basis of such ultrastable Brillouin laser references, a 10-GHz microwave oscillator with phase noise as low as −65 dBc (decibels relative to the carrier)/Hz at 1 Hz, −155 dBc/Hz at 10 kHz, and −172 dBc/Hz at 10 MHz is generated using a simple EOM comb divider with an unprecedentedly small division ratio of only 10, orders of magnitude smaller than prior OFD systems (see Table 1). This work redefines the trade-off between division complexity and noise suppression in OFD, enabling compact, affordable, deployable microwave synthesis with unrivaled spectral purity. Moreover, by leveraging the exceptional low-noise performance of the OFD signal as a reference, we have realized a broadband frequency synthesizer operating in the 5- to 20-GHz band, which outperforms state-of-the-art commercial signal generators across the offset frequency of 1 Hz to 1 MHz.

**Table 1.** Performance comparison of various 2P-OFD systems.

Optical references	Comb divider	Division ratio^*^	Single-sideband phase noise (dBc/Hz)^*^					Ref.
			1 Hz	100 Hz	1 kHz	10 kHz	1 MHz	
Brillouin lasers	EOM comb	Unspecified	/	−103	−128	−154	−157	(*10*)
Cavity-stabilized lasers	Microcomb	64	/	−102	−124	−141	−134	(*23*)
Cavity-stabilized lasers (vacuumed)	EOM comb	130	−60	−110	−136	−156	−133	(*24*)
Cavity-stabilized lasers	EOM comb	226	/	−43	−99	−141	−133	(*25*)
Cavity-stabilized lasers	Microcomb	300	/	−101	−133	−152	−136	(*26*)
Cavity-stabilized lasers	EOM comb	343	/	−94	−120	−144	−133	(*27*)
Brillouin lasers	Microcomb	360	/	−56	−81	−129	−130	(*28*)
Cavity-stabilized lasers	Microcomb	600	/	−86	−107	−134	−144	(*29*)
Brillouin lasers	EOM comb	2960	/	−110	−132	−142	−153	(*30*)
Cavity-stabilized lasers (vacuumed)	EOM comb	52	−82	−128	−141	−148	−128	(*31*)
Cavity-stabilized lasers	Microcomb	586	/	−104	−114	−142	−141	(*32*)
Cavity-stabilized lasers	Microcomb	250	/	−115	−131	−149	−151	(*33*)
Brillouin lasers	**EOM comb**	**10**	**−65**	**−109**	**−136**	**−155**	**−155**	**This work**

* The division ratio and single-sideband phase noise values are all scaled to the 10-GHz carrier for a direct comparison.

## RESULTS

### Generation of microhertz-linewidth two-color Brillouin lasers

**Fig. 2.**
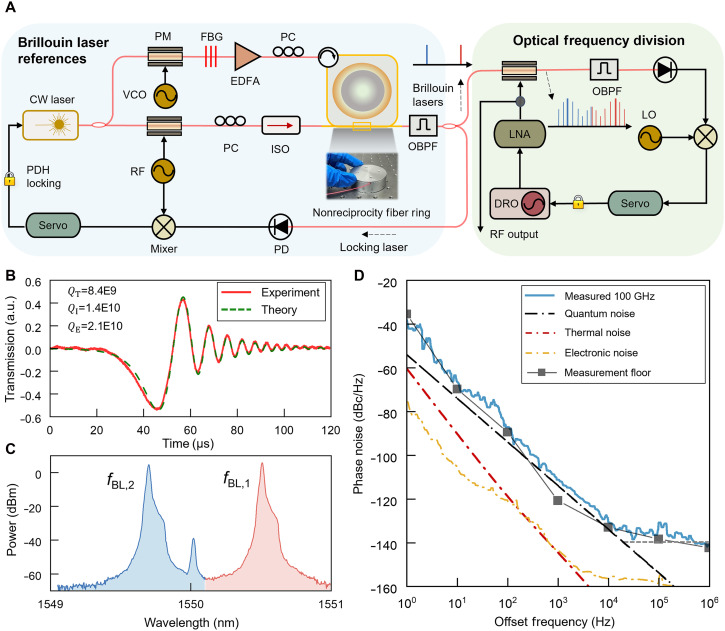
Generation and characterization of microhertz-linewidth Brillouin laser references. (**A**) Experimental setup. FBG, fiber Bragg grating; ISO, isolator; PC, polarization controller; PM, phase modulator; OBPF, optical bandpass filter; PD, photodiode; LNA, low-noise amplifier. (**B**) Ring-down test of the fiber cavity, from which *Q*-factors are theoretically extracted. a.u., arbitrary units. (**C**) Optical spectrum of the generated Brillouin lasers, whose frequency separation is 100 GHz, configured by selecting the second-order modulation sidebands from the 25-GHz VCO-driven phase modulator. (**D**) Measured phase noise of the 100-GHz beat note generated by the two-color Brillouin lasers within a uni-traveling-carrier photodiode, which approaches the measurement floor. The noise floor values are derived from the product manual of RS FSWP B61. Theoretical analysis reveals that the Brillouin lasers are quantum noise limited corresponding to the ST linewidth of 16.8 μHz. Thermal noise and electronic noise are suppressed below the quantum noise, as explained in the Supplementary Materials.

Figure 2A shows the experimental setup for Brillouin laser generation and OFD operation. A 200-m-long standard single-mode fiber spool, a 2 × 2 fiber coupler (95:5), and a three-port fiber circulator are used to build a nonreciprocal fiber-ring cavity. As shown in Fig. 2B, this fiber cavity has ultrahigh *Q*-factors approaching 10 billion, measured from the cavity ring-down waveform (*41*). The output of a continuous wave (CW) laser is split into two parts: One part is stabilized to a counterclockwise fiber cavity mode via PDH locking, and the other part is modulated using an electro-optical phase modulator. This phase modulator is driven by a 25-GHz voltage-controlled oscillator (VCO) to generate a series of modulation sidebands, from which two sidebands with 100-GHz separation are selected out using optical filters. After amplification by an erbium-doped fiber amplifier, the selected sidebands are injected into the fiber cavity in the clockwise direction, serving as pump lasers to stimulate the generation of two-color Brillouin lasers via stimulated Brillouin scattering gain, as shown in Fig. 2C. The Brillouin lasers are frequency blue-shifted by about 10.9 GHz from the pump lasers, and both have a power of ~3 mW. Notably, given that the CW laser is PDH locked to the fiber cavity resonance, the pump sidebands also remain frequency-stable relative to the fiber cavity modes, thus preventing mode hopping of the generated Brillouin lasers and ensuring long-term stability.

The generated two-color Brillouin lasers are detected using a uni-traveling-carrier photodiode, wherein a 100-GHz electrical beat note is generated. This beat note is then measured using a millimeter-wave (mmWave) phase noise analyzer (RS FSWP B61 Z110 module), and its phase noise reaches −111 dBc/Hz at 1 kHz and −131 dBc/Hz at 10 kHz, as shown in Fig. 2D (see Materials and Methods). Although some noise floor limitations are present in this region, the measured phase noise still clearly exhibits a characteristic −20 dB/decade slope until it intersects with the PD shot-noise floor, indicating that it is dominated by the quantum noise of the Brillouin lasers. Theoretically, the contribution of quantum noise to the mutual phase noise between the Brillouin lasers can be expressed as (*42*, *43*)

$$∆S_{\text{quantum}}\left(f\right)=f^{-2}\frac{h\left(f_{\text{BL},1}^{3}+f_{\text{BL},2}^{3}\right)n_{\text{th}}}{2Q_{T}Q_{E}P_{B}}$$ (4)

*h* is the Planck constant; $P_{B}$, $f_{\text{BL},1}$, and $f_{\text{BL},2}$ are the output power and frequencies of the generated Brillouin lasers, respectively; $n_{\text{th}}$ is the thermal occupation of the phonon mode; and $Q_{T}$ and $Q_{E}$ denote the loaded and external *Q*-factors of the laser cavity, respectively. Fitting Eq. 4 to the experimental data yields a fundamental ST linewidth of 16.8 μHz for both Brillouin lasers, enabled by the ultrahigh $Q_{T}$ and $Q_{E}$ of the fiber ring cavity (see the Supplementary Materials). When these microhertz-linewidth Brillouin lasers are used as the optical references (i.e., $f_{\text{BL},1}=f_{\text{REF}1}$ and $f_{\text{BL},2}=f_{\text{REF}2}$), they bring about extremely low level $∆S_{\text{REF}}$ (see Fig. 2D) that facilitates efficient OFD with a minimal division ratio.

It is important to scrutinize and confirm that the contributions from thermal noise and electronic noise in our system are below the quantum noise of the Brillouin lasers. First, given that the two-color Brillouin lasers are generated within the same fiber cavity and subjected to CMNR, their mutual phase noise caused by cavity thermal fluctuations ($∆S_{\text{thermal}}$) is significantly reduced compared to the intrinsic thermal noise of each individual laser ($S_{T}$); such suppression can be quantified as (*31*)

$$∆S_{\text{thermal}}\left(f\right)={\left(\frac{f_{\text{BL},1}-f_{\text{BL},2}}{f_{\text{BL},1}}\right)}^{2}S_{T}\left(f\right)$$ (5)

In our experiment, $\left(f_{\text{BL},1}-f_{\text{BL},2}\right)/f_{\text{BL},1}∼$ 1/1935, meaning that $∆S_{\text{thermal}}$ is about 66 dB smaller than $S_{T}\left(f\right)$. As shown in Fig. 2D, by estimating $S_{T}\left(f\right)$ using the Duan and Wanser models (see the Supplementary Materials) (*44*, *45*), the differential thermal noise $∆S_{\text{thermal}}$ lies far below the quantum noise of Brillouin lasers. It is noteworthy that by substituting Eq. 1 into Eq. 5, we have

$$∆S_{\text{thermal}}\left(f\right)∼N^{2}{\left(\frac{f_{\text{spc}}}{f_{\text{BL},1}}\right)}^{2}S_{T}\left(f\right)$$ (6)

Equation 6 indicates that for 2P-OFD, the mutual thermal noise $∆S_{\text{thermal}}$ between two optical references increases with $N^{2}$, meaning that $∆S_{\text{thermal}}$ cannot be divided by implementing bigger *N*. Instead, it is fundamentally constrained by the intrinsic thermal noise $S_{T}$ of the reference platform at the frequency $f_{\text{BL},1}$, which in turn sets the ultimate noise limit of an OFD system (*31*, *46*).

Second, in our system, the CW laser is PDH locked to the fiber cavity mode, during which electronic noise is inevitably introduced. Nevertheless, the pump lasers that actually generate the Brillouin lasers are modulation sidebands, between which the electronic noises added by the PDH locking loop are correlated and thus cancelled out. Instead, the dominant phase noise between the pump sidebands is from the 25-GHz driving VCO, which is much lower than the locking electronic noises (see the Supplementary Materials). Furthermore, during the generation of Brillouin lasers, the pump lasers’ phase noises added by the 25-GHz VCO are markedly suppressed through cavity filtering and phase damping induced by the acoustic wave (*47*, *48*). These combined effects reduce the electronic noise contribution ($∆S_{\text{electronic}}$) to a negligible level compared to the Brillouin lasers’ quantum noise floor, as shown in Fig. 2D (see the Supplementary Materials for detailed experimental and theoretical analysis).

### Demonstration of OFD referenced to microhertz-linewidth Brillouin lasers

**Fig. 3.**
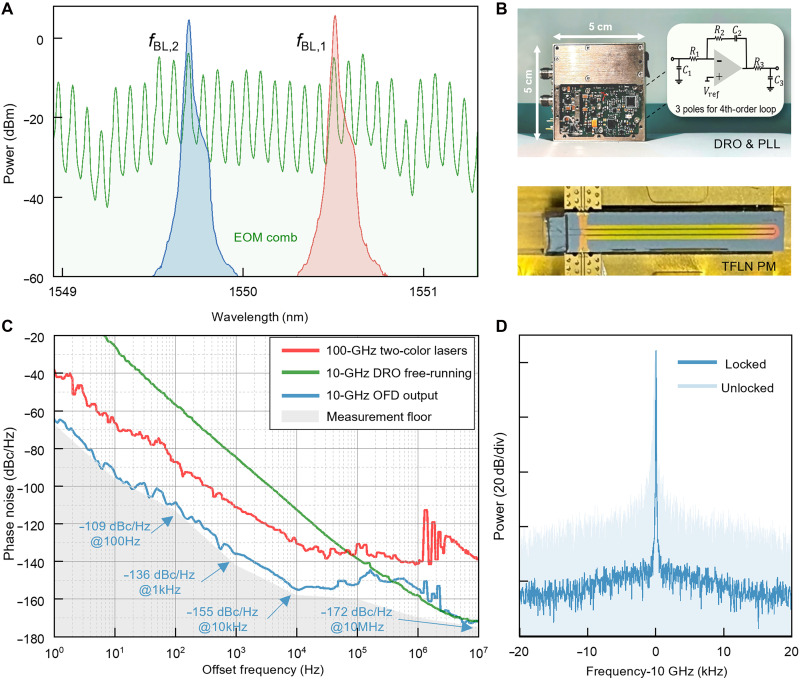
Demonstration of 2P-OFD referenced to microhertz-linewidth Brillouin lasers. (**A**) Optical spectrum of the Brillouin laser references and the EOM comb divider. The fifth-order modulation sidebands from both combs overlap, correspondingly *N* = 10, and the OFD output frequency *f*_OFD_ is 10 GHz. (**B**) Picture of the DRO module, the PLL circuits, and the TFLN phase modulator used for OFD implementation. (**C**) Measured phase noises of the free-running 10-GHz DRO signal and the OFD output signal after the PLL is closed. The measurement floor illustrated in shadow is derived from the product manual of RS FSWP B61. (**D**) Electrical spectrum of the 10-GHz signal before and after the PLL is closed.

The detailed experimental setup to implement OFD is illustrated in Fig. 2A. The generated two-color Brillouin lasers, serving as the optical references, are combined and modulated using a thin-film lithium niobate (TFLN) optical phase modulator. The phase modulator is driven by a 10-GHz dielectric resonator oscillator (DRO), which is power boosted to 27 dBm using an ultralow-noise electrical amplifier, so that two EOM combs with fifth-order modulation sidebands (i.e., division ratio *N* = 10) are generated from both Brillouin lasers to close up the 100-GHz gap between them and produce an IF beat note, as shown in Fig. 3A. The IF beat note is down-mixed to produce the OFD error signal, which is sent into an optimized phase-locked loop (PLL; see Fig. 3B) to feedback control the DRO. The DRO output frequency is tunable within a range of 10 MHz, and the voltage tuning coefficient and bandwidth are 0.8 MHz/V and 5 MHz, respectively. The PLL uses a typical three-pole, fourth-order loop filter, with its bandwidth and phase margin carefully optimized to synergistically combine the low-phase-noise performance of the DRO (>200-kHz offset) and the divided differential phase noise Δ*S*_REF_ of the Brillouin laser references (<200-kHz offset) (*40*). Notably, the noise bump observed in the 100-GHz beat note between 1 and 10 MHz, which is attributed to the residual side modes of the Brillouin lasers, lies well outside the bandwidth of the PLL. It is therefore effectively suppressed and does not degrade the phase noise of the 10-GHz output signal.

After the OFD loop is closed, the 10-GHz DRO signal illustrates a substantially narrowed spectrum than the full-running state (see Fig. 3D), and its phase noise is measured as low as −65 dBc/Hz at 1 Hz, −136 dBc/Hz at 1 kHz, −155 dBc/Hz at 10 kHz, and −152 dBc/Hz at 100 kHz (see Fig. 3C), corresponding to a root-mean-square time jitter of 1.15 fs (integrated from 10 kHz to 80 MHz). Compared to the 100-GHz phase noise curve ($∆S_{\text{REF}}$) between the referenced Brillouin lasers, the 10-GHz signal achieves ~20-dB (i.e., *N*^2^ = 100) noise suppression ranging from 10 Hz to 200 kHz. From 200 kHz to 1 MHz, the PLL achieves a smooth transition to the intrinsic DRO phase noise without large peaking, ultimately reaching down to −172 dBc/Hz at 10-MHz offset (*40*).

### Phase noise analysis of the OFD system

**Fig. 4.**
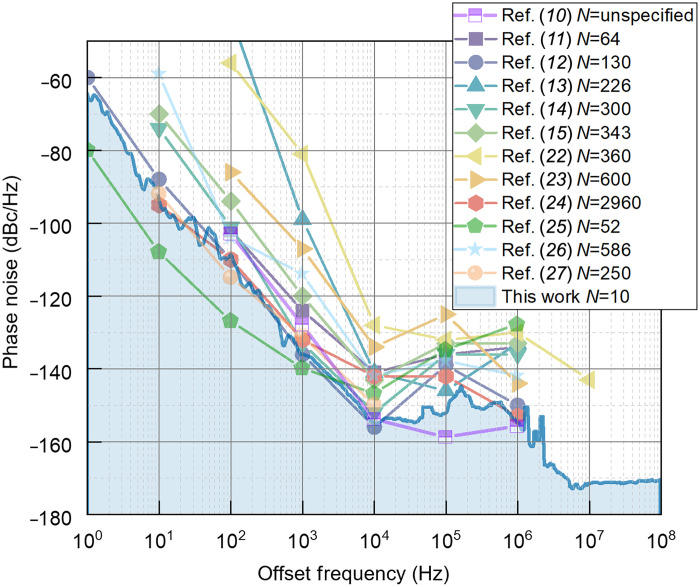
Comparison of phase noises for various 2P-OFD architectures. For comparison, the division ratio *N* and phase noise traces are scaled to the 10-GHz carrier frequency. Detailed configurations used in the references are described in Table 1.

Figure 4 and Table 1 compare our OFD system with other OFD architectures. Despite using the smallest division ratio (*N* = 10) reported to date, our system achieves phase noise and time jitter metrics that outperform most of the prior OFD implementations. The key to this achievement is the generation of microhertz-linewidth quantum noise–limited Brillouin laser references based upon the combination effects including the following: (i) CMNR of cavity thermal fluctuations; (ii) coherent sideband pumping that cancels the PDH locking electronic noise, especially the servo pumps at a high offset frequency; (iii) acoustic wave–induced phase noise damping during stimulated Brillouin scattering; and (iv) ultrahigh *Q*-factor of the fiber cavity that quadratically reduces the ST linewidths of the Brillouin lasers.

### OFD-based broadband frequency synthesizer

**Fig. 5.**
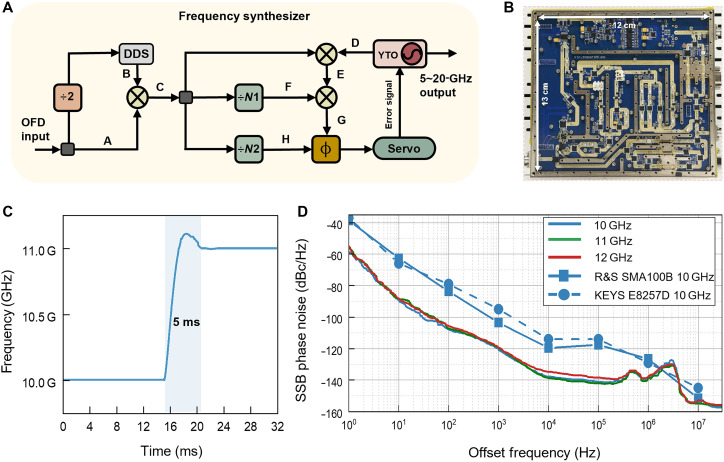
Wideband frequency synthesizer based on the OFD signal. (**A**) System architecture diagram of the frequency synthesizer. “÷2,” “÷*N*1,” and “÷*N*2” all denote dividers; ϕ, phase detector. (**B**) Hardware module of the frequency synthesizer, with dimensions of 12 cm by 13 cm. (**C**) Frequency hopping time test of the wideband frequency synthesizer. The output frequency switches from 10 to 11 G, tested using the phase noise transient analysis function of the R&S FSWP. (**D**) The phase noise of the frequency synthesizer’s output signal presents performance at three frequency points (5 G: blue line; 10 G: green line; 20 G: purple line) and is compared with the R&S SMA100B B709 option (blue squares) and Keysight E8257D HY2 option (blue circles). SSB, single sideband.

To fully harness the exceptional spectral purity of the OFD-generated microwave signal, it needs to serve as the foundational reference for broadband, agile frequency synthesis across a wide operational range. In this section, we demonstrate an ultralow-noise broadband frequency synthesizer that integrates the OFD-generated 10-GHz reference with a direct digital synthesizer (DDS) and a PLL architecture. As illustrated in Fig. 5 (A and B), the OFD signal serves as both the primary reference and clock source for the DDS (after a divide-by-two stage to 5 GHz). To mitigate the DDS’s inherent noise rises at higher frequencies imposed by the Nyquist sampling limit, the DDS output is limited to around 500 MHz, minimizing its contribution to the wideband noise floor. This low-noise DDS signal is then mixed with the OFD output to produce a clean, tunable 10.5-GHz reference.

The system uses a YIG (yttrium, iron, garnet)–tuned oscillator (YTO) to achieve wideband coverage from 5 to 20 GHz, with microhertz resolution and a speed of 5 ms, as shown in Fig. 5C. The 10.5-GHz reference is down-converted through a two-stage mixing loop, and the YTO is phase locked to a derivative of this reference via a phase detector. For example, when generating a 12-GHz output, the signal frequencies at key nodes are as follows: A: 10 GHz (OFD reference); B: 549 MHz (DDS output); C: 10.549 GHz (mixed reference); D: 12 GHz (YTO output); E: 1.451 GHz; F: 1.978 GHz; G: 527 MHz; H: 2.637 GHz. Critically, the ultralow phase noise of the OFD reference is preserved throughout the synthesis chain. As shown in Fig. 5D, the synthesized signals exhibit exceptional spectral purity; the 10-GHz (5 GHz, 20 GHz) signals from our system reach −121 dBc/Hz and −138 dBc/Hz (−126 dBc/Hz and −138 dBc/Hz, −114 dBc/Hz and −132 dBc/Hz) at 1- and 10-kHz offsets, respectively. These results outperform state-of-the-art commercial signal generators (R&S SMA100B B709 low-noise option and Keysight E8257D HY2 option) by >15 dB in the 1-Hz to 100-kHz offset range, precisely where phase noise critically affects radar resolution, communication fidelity, and metrological stability. This demonstrates that our OFD-based synthesizer not only achieves broad tunability but also sets a benchmark for low-noise microwave generation across multioctave bandwidths.

## DISCUSSION

To make OFD a broadly deployable technique, it is vital to minimize its form factor and complexity to rival those electronic counterparts. The present work marks solid advances toward this goal. The EOM comb divider with *N* = 10 is markedly simplified compared to prior structures (e.g., femtosecond laser comb, Kerr microcomb, and cascaded EOM comb) and able to be readily fabricated on a TFLN wafer, together with the modulators used for PDH locking and sideband generation (*49*, *50*) and the passive optical components (filters, splitters, etc.) (*51*). The single CW laser and optical amplifiers used in our system can also be built on-chip via heterogeneous or hybrid integration that is under fast development (*52*, *53*). The electronic control circuits are inherently compact (see Fig. 3B). Meanwhile, the 200-m-long fiber cavity, which is the bulkiest part in our OFD system, has a volume of just 40 ml (*48*). This fiber cavity is marginally larger than the miniaturized FP cavity (*23*) and on-chip coil-reference cavity (*25*), but it needs no alignment and mode conversion, features much better robustness, supports full polarization maintenance, offers much higher *Q*-factor, has much lower thermal noise limit, demands much less fabrication cost, and enables quantum-limited microhertz-linewidth Brillouin lasers with unrivaled low $∆S_{\text{REF}}$. So, we argue that fiber-cavity Brillouin lasers could by far be the optimal reference for compact and deployable OFD.

Last, according to Eqs. 2 and 6, the ultimate noise limit of an OFD system ($S_{\text{lim}}$) can be derived as

$$S_{\text{lim}}\left(f\right)∼{\left(\frac{f_{\text{spc}}}{f_{\text{BL},1}}\right)}^{2}S_{T}\left(f\right)$$ (7)

For our OFD system referenced to the fiber Brillouin lasers, $S_{T}$ consists of the intrinsic thermal conductive noise and thermal mechanical noise of the fiber cavity (*54*, *55*). As elucidated in the Supplementary Materials, for the 200-m-long single-mode fiber cavity (placed in a simple air-tight box), $S_{T}$ is about 0 dBc/Hz at 1-Hz offset and −80 dBc/Hz at 1-kHz offset when $f_{\text{BL},1}$ ~ 193.5 THz (*56*). Correspondingly, our OFD system has a projected noise limit $S_{\text{lim}}$ down to −85 dBc/Hz at 1-Hz offset and −165 dBc/Hz at 1-kHz offset for a 10-GHz oscillator ($f_{\text{spc}}$ = 10 GHz), requiring a division ratio of ~100 that can be implemented using state-of-the-art TFLN modulators (*25*, *27*) or Kerr microcombs (*23*, *26*, *28*, *29*, *32*, *33*). Notably, the estimated phase noise limit of our scheme is comparable with the record-setting 1f-2f OFD built with vacuum-enclosed ultrahigh-finesse reference cavity, octave-spanning femtosecond laser comb, superprecision pulse interleaving, highly linear pulse photodetection, and sophisticated control electronics (*12*, *22*), showcasing the potential of our scheme to redefine the performance, complexity, and practicality of the OFD system.

## MATERIALS AND METHODS

### Experiment setup of two-color Brillouin lasers and OFD operation

A standard single-mode fiber ring, a 2 × 2 fiber coupler (95:5), and a three-port fiber circulator are used to form an ultrahigh-*Q* optical resonator, which is crucial for achieving a low-noise bichromatic Brillouin laser oscillator (*40*). A CW laser is then PDH locked to the fiber cavity (counterclockwise) to ensure alignment with the cavity mode and preventing mode hopping of the backward-stimulated Brillouin laser. The pump laser is phase modulated by VCO to generate tunable dual-frequency pump laser tones. After amplification by an erbium-doped fiber amplifier, the pump tones are injected into the cavity (clockwise) to induce bichromatic stimulated Brillouin lasers. Figure 2A shows the detailed diagram of the generated two-color Brillouin lasers separated by around 100 GHz. The generated two-color Brillouin lasers are then combined and modulated using an optical phase modulator. The phase modulator is driven by a 10-GHz DRO, which is amplified to 27 dBm using an ultralow-noise electrical amplifier, to generate two EOM combs with fifth-order modulation sidebands. These sidebands bridge the 100-GHz gap between the original lasers, producing an IF beat note. The IF beat note is down-mixed to produce the OFD error signal, which is sent into an optimized PLL to feedback control the DRO.

### Phase noise measurement of the 100-GHz mmWave signal

Traditionally, phase noise measurement of a signal under test is limited to frequencies below 50 GHz because of instrument constraints. To extend measurements to mmWaves, an external harmonic mixer is used. The mixer down-converts the mmWave signal under test multiplied by a local oscillator (LO) signal to an IF. However, this process also generates abundant unwanted harmonics. To suppress phase noise from these LO harmonics and overcome the system’s sensitivity limitation imposed by LO phase noise, two external harmonic mixers enable cross-correlation. The 100-GHz signal first passes through a waveguide splitter and then connects to two external harmonic mixers. Two internal reference sources from the phase noise analyzer (R&S FSWP) function as LOs (LO1 and LO2). Each mixer down-converts the mmWave signal to an IF in the several megahertz range. Subsequent digital processing further down-converts this IF to 0 Hz. The resulting signal is divided into two ways to be used as the I/Q (in-phase/quadrature) signal. Cross-correlation and mmWave phase noise trace calculation are performed on a PC processor interfaced with the field-programmable gate array. Figure 2D shows the measured single-sideband phase noise of our 100-GHz mmWave, exhibiting −111 dBc/Hz at 1-kHz offset and −131 dBc/Hz at 10-kHz offset.

## Supplementary Materials

### This PDF file includes:

Supplementary Text S1 to S5 Figs. S1 to S6 Table S1 References
