# Supplementary material for: Superefficient optical frequency division referenced to μHz Schawlow-Townes-linewidth quantum noise–limited lasers
Source: Sci Adv. 2026 Jun 17;12(25):eaed1856. doi: 10.1126/sciadv.aed1856 (PMC13274596; doi:10.1126/sciadv.aed1856)
Supplement: Supplementary file 1 — Supplementary Text S1 to S5 Figs. S1 to S6 Table S1 References [file sciadv.aed1856_sm.pdf]

Science Advances
AAAS

Supplementary Materials for

Superefficient optical frequency division referenced to μHz Schawlow-Townes-linewidth quantum noise–limited lasers

Jiahao Hu *et al.*

Corresponding author: Jing Xu, jing_xu@hust.edu.cn; Huashun Wen, whs@nankai.edu.cn;
Heng Zhou, zhouheng@uestc.edu.cn

Sci. Adv. **12**, eaed1856 (2026)
DOI: 10.1126/sciadv.aed1856

This PDF file includes:

Supplementary Text S1 to S5
Figs. S1 to S6
Table S1
References

Supplementary Text

S1. Theoretical derivation of the two-color Brillouin lasers phase noise

In this section, we represent detailed theoretical derivation of the phase noise mechanisms between the two-color Brillouin lasers. First, a continuous-wave (CW) laser is modulated by an electro-optical phase modulator, the output field can be written as:

$$E_{\mathrm{MOD},p}(t) = A_{\mathrm{MOD},p}\sin[(\omega_{cw} + p\omega_{VCO})t + \varphi_{cw}(t) + p\varphi_{VCO} + \theta], \theta = \begin{pmatrix} \frac{\pi}{2}, p = 0, \pm 2 \ldots \\ 0\ , p = \pm 1, \pm 3 \ldots \end{pmatrix} \quad \text{(S1)}$$

$$A_{\mathrm{MOD},p} = \begin{pmatrix} E_{\mathrm{c}}(-1)^{|p|/2} J_{|p|}(\beta), |p| = 0,2,4,6 \ldots \\ E_{\mathrm{c}}(-1)^{(|p|+1)/2} J_{|p|}(\beta), |p| = 1,3,5,7 \ldots \end{pmatrix} \quad \text{(S2)}$$

ω_{cw} and φ_{cw} denote the frequency and phase of the original CW laser. φ_{VCO} denotes the phase variation of the 25 GHz driving signal generated by the voltage controlled oscillator (VCO). $\beta = \pi V_{\mathrm{elec}}/V_{\pi}$ denotes the modulation index, which depends on the modulator V_{π} and applied electrical voltage V_{elec}. J_p is the pth order Bessel function of the first kind. As mentioned in the main text, the second order sidebands separated by 100 GHz (i.e., p=2) are selected as pump lasers for Brillouin laser generation. The optical fields of the generated Brillouin lasers can be expressed as (*57*):

$$E_{\mathrm{BL},m}(t) = A_{\mathrm{BL},m}\cos\left[(\omega_c \pm 2\pi \cdot 10^{11} + \Omega_m)t + \varphi_{\mathrm{Q},m}(t) + \varphi_{\mathrm{P},m}(t) + \varphi_{\mathrm{T},m}(t)\right] \quad \text{(S3)}$$

Here m =1 or 2. $A_{\mathrm{BL},m}$ represents the Brillouin laser amplitude, whose analytical expression can be found in Ref. (*58*). Ω_m donates the Brillouin frequency shift. $\varphi_{\mathrm{Q},m}$ is the quantum phase variation of the Brillouin lasers, $\varphi_{\mathrm{P},m}$ is the transferred phase variation from the pump lasers, $\varphi_{\mathrm{T},m}$ denotes the Brillouin laser phase noise caused by fiber thermal noise. The power spectrum density (PSD) of the phase noise between the two-color Brillouin lasers $S_{\mathrm{BL}}(t)$ can be expressed as:

$$S_{\mathrm{BL},}(f) = \Delta S_{\mathrm{quantum}}(f) + \Delta S_{\mathrm{electronics}}(f) + \Delta S_{\mathrm{thermal}}(f) \quad \text{(S4)}$$

$S_{\mathrm{quantum}}(f)$ denotes the relative phase noise PSD between the bi-chromatic Brillouin lasers caused by the fundamental quantum noise . Since the quantum-limited statistic phase variations $\varphi_{\mathrm{Q},m}$ are independent in nature (*59*), according to Wiener-Khinchin theorem we have:

$$\Delta S_{\mathrm{quantum}}(f) = S_{\mathrm{quantum,BL1}} + S_{\mathrm{quantum,BL2}} = f^{-2}\frac{h(f_{\mathrm{BL,1}}^3 + f_{\mathrm{BL,2}}^3)n_{\mathrm{th}}}{2Q_{\mathrm{T}}Q_{\mathrm{E}}P_{\mathrm{B}}} \quad \text{(S5)}$$

$f_{\mathrm{BL},m} = (\omega_c \pm 2\pi 10^{11} + \Omega_m)/2\pi$ denotes the center frequency of the Brillouin lasers. In Eq. (S5) we assume that the Q-factors ($Q_\mathrm{T}, Q_\mathrm{E}$) and output power ($P_\mathrm{B}$) are identical for the two Brillouin lasers (approximating our experiment condition).

Brillouin laser field also contains phase fluctuations transferred from the pump laser ($\varphi_{\mathrm{P},m}$), which contains the electronic noise induced during PDH locking and the VCO noise during phase modulation. When using modulation sidebands as the pump lasers, the transferred phase fluctuations onto each Brillouin laser can be expressed as:

$$\varphi_{\mathrm{TBL},m}(t) = \left(\frac{\kappa_m}{\kappa_m+\Gamma}\right)\varphi_{\mathrm{P},m}(t) = \left(\frac{\kappa_m}{\kappa_m+\Gamma}\right)\left[\varphi_{\mathrm{cw}}(t) + 2\varphi_{\mathrm{VCO}}(t)\right] \quad \text{(S6)}$$

Here $\kappa_m = 2\pi f_{\mathrm{BL},m}/Q_\mathrm{T}$ and Γ is the Brillouin gain linewidth. Eq. (S6) shows that during the generation of Brillouin lasers, the pump laser phase noises are suppressed by $\kappa_m/(\kappa_m + \Gamma)$, through cavity resonance filtering and phase damping induced by the acoustic wave (*47*, *48*). For the bi-chromatic Brillouin laser generated in the same cavity, we can assume κ_m is identical for both Brillouin lasers ($\kappa_1 = \kappa_2 = \kappa$), and their phase noises induced by the CW laser phase jitter φ_{cw}, which is dominated by the PDH locking electronic noise (*31*), are correlated and cancelled out, so the residual electronic noise is primarily from the 25 GHz VCO noise S_{VCO}:

$$\Delta S_{\mathrm{electronics}}(f) = 16 \times \left(\frac{\kappa}{\kappa+\Gamma}\right)^2 S_{\mathrm{VCO}}(f) \quad \text{(S7)}$$

Using the experimental measured Q-factor $Q_T = 8.4 \times 10^9$ and the typical Brillouin gain linewidth in silica fiber $\Gamma = 2\pi \cdot 30$ MHz, we can calculate that $(\kappa/\kappa + \Gamma)^2$ is about -60.5 dB. Consequently, the phase noise transferred from the VCO becomes insignificant (see Fig. S1A).

In Eq. S3, $\varphi_{\mathrm{T},m}(t)$ denote the Brillouin laser phase variations caused by fiber thermal noise. Based on the Duan and Wanser model (*44*, *45*), the phase noise PSD of the cavity resonance frequency caused by the fiber thermal noise can be expressed as:

$$S_\mathrm{T}(f) = S_{\mathrm{TC}}(f) + S_{\mathrm{TM}}(f) \quad \text{(S8)}$$

The thermo-conductive noise term $S_{\mathrm{TC}}(f)$ can be estimated using Wanser's model (*45*):

$$S_{\mathrm{TC}}(f) = \frac{1}{f^2}\left(f_{\mathrm{BL},m}\frac{1}{n}\frac{dn}{dT}\right)^2 \frac{k_B T^2}{2\pi\kappa L}\ln\left(\frac{k_{max}^4+\left(\frac{2\pi f}{D}\right)^2}{k_{min}^4+\left(\frac{2\pi f}{D}\right)^2}\right) \quad \text{(S9)}$$

dn/dT is the refractive index temperature coefficient of silica fiber, k_B is the Boltzmann constant, T is the temperature, κ is the thermal conductivity, L is the length of the fiber cavity, k_{max} and

k_{min} are the boundary conditions and D is the thermal diffusivity (parameters are listed in Table S1 and the calculated results derived from their substitution are subsequently plotted in Fig. S1B.). The thermo-mechanical noise term $S_{\mathrm{TM}}(f)$ can be estimated using Duan's model (*44*):

$$S_{\mathrm{TM}}(f) = \frac{1}{f^3}\left(\frac{f_{\mathrm{BL},m}}{L}\right)^2 \frac{2k_B T L \phi_0}{3\pi A E_0} \tag{S10}$$

ϕ_0 is the loss angle that characterizes mechanical dissipation in single mode fiber, A is the cross-sectional area of the fiber and E_0 is the bulk modulus of silica.

For two-color Brillouin lasers generated within the same fiber cavity, their relative phase noise is subject to common-mode noise rejection (assuming that each Brillouin laser frequency follows the corresponding resonance frequency) and determined by the cavity's differential thermal noise (*31*):

$$\Delta S_{\mathrm{thermal}}(f) = \left(\frac{f_{\mathrm{BL},1} - f_{\mathrm{BL},2}}{f_{\mathrm{BL},1}}\right)^2 S_{\mathrm{T}}(f) \tag{S11}$$

S2. Experimental investigation of the two-color Brillouin lasers phase noise

After the Brillouin lasers are generated, they are detected using a uni-traveling-carrier photodiode (UTC-PD), wherein a 100 GHz electrical beat note is generated, and then measured using an mmWave phase noise analyzer (RS FSWP B61 Z110 module), as shown in Fig. S2. Based on Eq. (S5), to minimize the quantum noise term $\Delta S_{\mathrm{quantum}}$ of the Brillouin lasers, we optimize the Q-factors of the fiber cavity and maximize the Brillouin laser output power. As shown in Fig. 2B and 2C in the main text, Q-factor of our specifically-designed fiber cavity is about 10 billion (Q_{T}=8.4 billion, and Q_{E}=21 billion), and the output power P_{B} is set at 3 mW.

Theoretically modeled phase noise contributions using Eqs. (S5, S7, S8) are illustrated in Fig. S2. It is seen that the experimentally measured phase noise curve matches well with the quantum noise contribution, confirming that the Brillouin lasers operate in the quantum-noise-limited state. The fundamental Schawlow-Townes linewidth Δv_{ST} of both Brillouin laser is related to the quantum phase noise via the following relation (*43*):

$$\Delta v_{\mathrm{ST}} = 2\pi \cdot f^2 S_{\mathrm{quantum,BL}} \tag{S12}$$

Substituting the experimental parameters into Eqs. (S5, S12) (see Table S1) and assuming the two-color Brillouin lasers have identical fundamental linewidth and contribute equally to the measured phase noise, we can obtain that the fundamental linewidth for both Brillouin lasers is Δv_{ST}= 16.8 μHz, corresponding to a quantum-limited white frequency noise floor of 2.67×10^{-6} Hz^2/Hz. These measurements are consistent with our recent publication (*40*).

In contrast, classical noise sources—electronic noise in the stabilization loops and cavity thermal noise—are suppressed to negligible levels, as shown in Fig. S2. Above 10 kHz, the phase noise converges to the UTC-PD shot-noise floor, which does not affect OFD performance since the UTC-PD is absent during the division process.

S3. Fractional frequency instability of the OFD 10 GHz signal

To assess the long-term stability of the synthesized microwave signal, we measured the Allan deviation of the 10 GHz OFD output. As shown in Fig. S3, the fractional frequency stability of the system, measured using the Allan Deviation function of the R&S FSWP phase noise analyzer, reaches 2.4×10^{-11} at 1 s averaging time. The FSWP analyzer (equipped with the B61 model) measures Allan deviation by directly analyzing the phase fluctuations of the input microwave signal using high-resolution internal digitization techniques. This enables highly sensitive stability measurements down to a fractional frequency instability of approximately 1×10^{-13} at 1 s for 10 GHz signals, limited primarily by the instrument's internal reference stability. In our setup, the measured Allan deviation of 2.4×10^{-11} at 1 s is dominated by environmental perturbations of the fiber cavity, not by the analyzer's sensitivity. The fiber cavity was housed in a passive aluminum enclosure during the measurement; further improvements in stability could be realized with active environmental control or by referencing the output to an atomic clock, though such optimizations are outside the scope of this study.

S4. Relevant demonstration of the frequency division factor *N* = 20

The detailed experimental setup to implement *N* = 20 OFD is the same as Fig. 2A. The generated two-color Brillouin lasers, serving as the optical references, are combined and modulated using an

TFLN optical phase modulator. The phase modulator is driven by a 10 GHz dielectric resonator oscillator (DRO), which is power boosted to 30 dBm using a high-power electrical amplifier, so that two EOM combs with 10th order modulation sidebands (i.e., division ratio N=20) are generated from both Brillouin lasers to close up the 200 GHz gap between them and produce an intermediate frequency (IF) beat note, as shown in Fig. S4A. The IF beat note is down-mixed to produce the OFD error signal, which is sent into an optimized phase-lock-loop (PLL, see Fig. 3B) to feedback control the DRO.

After the OFD loop is closed, the 10 GHz OFD signal measured phase noise is as low as -113 dBc/Hz at 100 Hz, -140 dBc/Hz at 1 kHz and -152 dBc/Hz at 10 kHz (see Fig. S4B). It should be noted that the PLL parameters have been adjusted accordingly for the division factor of N = 20. This adjustment results in a flat trend in the phase noise performance of the OFD signal between 30 kHz and 400 kHz, which differs significantly from that of the N = 10 OFD system presented in the main text Fig. 4. Compared with our frequency division results when N = 10, this performance level shows no significant improvement at offset frequencies around 10 kHz. That is to say, achieving the full benefit of a large division ratio would thus require a complete redesign of the phase-locking electronics including low-noise mixers, phase detectors, and loop filters, which is a significant undertaking beyond the scope of this manuscript. Nevertheless, we emphasize that with N = 10, our system already generates top-notch low phase noise that meets the requirements of most microwave applications.

S5. Testing of the OFD-Based Broadband Frequency Synthesizer

In the initial manuscript Fig. 5C, we demonstrated the frequency hopping performance of our OFD-based broadband frequency synthesizer from 10 GHz to 11 GHz, showing a millisecond-level tuning time. To address the question of whether such rapid tuning can be achieved across the entire 5–20 GHz operating range, we provide additional experimental data and analysis here.

We tested the small-step frequency tuning from 5 GHz to 5.08 GHz using the PN transient analysis function of a phase noise analyzer, as shown in Fig. S5A. The measured hopping time is consistent with the result obtained for the 10 GHz to 11 GHz case presented in the initial manuscript. Due to the bandwidth limitation of the phase noise analyzer in measuring frequency

hopping time, we adopted an alternative method to evaluate the broadband frequency tuning time. Taking the 10–14 GHz frequency hopping shown in Fig. S5B as an example, we implemented the measurement using a low-speed oscilloscope with a 100 MHz bandwidth and a mixer. The 10 GHz output signal from our frequency synthesizer served as the initial LO signal for the mixer, which was mixed with an external RF source operating at 14.01 GHz to generate a 4.01 GHz IF signal, fed into CH1 of the oscilloscope. As this signal far exceeded the oscilloscope's bandwidth, the oscilloscope effectively acted as a low-pass filter, displaying only a flat line. Subsequently, the host computer controlled the frequency synthesizer (with the control signal serving as the trigger signal on CH2 of the oscilloscope) to switch the output frequency to 14 GHz. At this point, the mixer's IF signal became 10 MHz, which fell within the oscilloscope's bandwidth and appeared as a sine wave. Using this method, we measured the frequency hopping time from 10 GHz to 14 GHz to be 6.2 ms, also remaining at the millisecond level.

In Fig. 5D of the initial main text, we demonstrated the phase noise performance of our OFD-based broadband frequency synthesizer at three representative frequencies (10 GHz, 11 GHz, and 12 GHz) to enable a direct comparison with state-of-the-art commercial signal generators (R&S SMA100B B709 and Keysight E8257D HY2). As suggested by the reviewer, to illustrate whether synthesized signals across the entire 5–20 GHz operating range can maintain low phase noise performance of our OFD system, we have provided additional phase noise measurements in Fig. S6 of the revised manuscript.

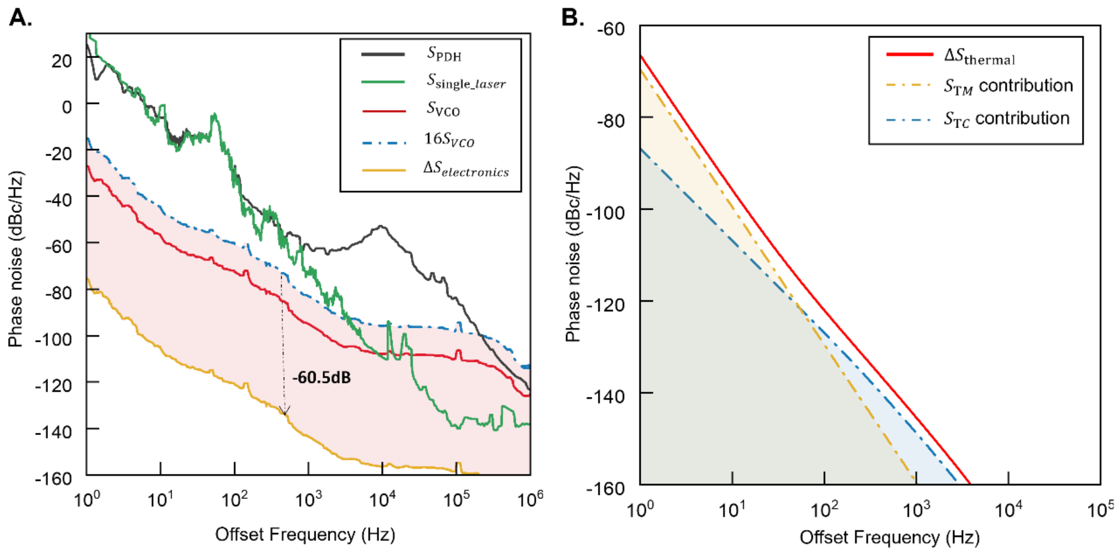

Fig. S1. Analysis of two-color Brillouin laser phase noise. (A) The electronic noise induced during PDH locking and the VCO noise during phase modulation. Following 16× amplification (blue line) and the pump laser phase noises suppression (yellow line), the Voltage-Controlled Oscillator (VCO) noise contribution achieves an ultra-low phase noise level, rendering it negligible in the overall system performance evaluation. S_{PDH} and $S_{\mathrm{single\_laser}}$ denote the phase noise of the PDH-locked CW laser and that of a single Brillouin laser, respectively. **(B)** The frequency noise of the cavity resonance frequency caused by the fiber thermal noise. At low frequency offsets, thermo-conductive noise term $S_{\mathrm{TC}}(f)$ is predominant, while at high frequency offsets, the thermo-mechanical noise term $S_{\mathrm{TM}}(f)$ becomes dominant.

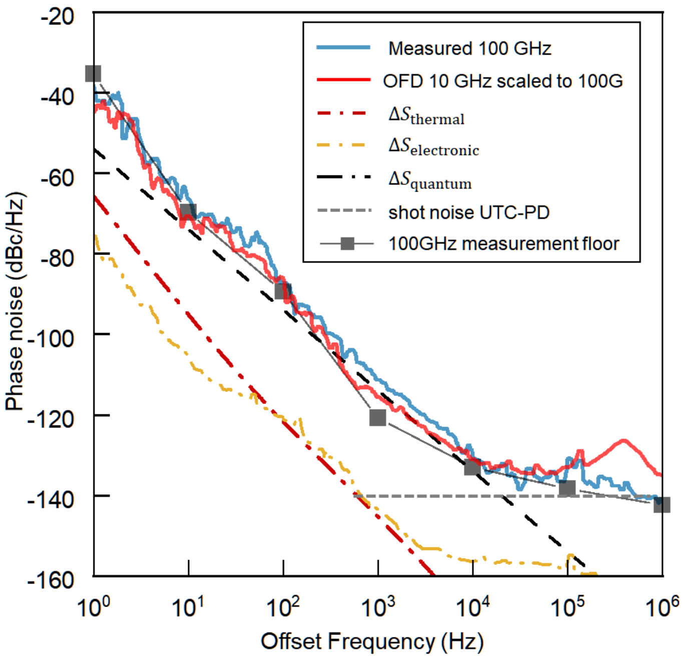

Fig. S2. The phase noise comparison of 100 GHz two-color lasers and different noise term. It can be found that the 100 G signal is primarily limited by the quantum noise and shot noise of the uni-traveling-carrier photodiode (UTC-PD). Due to the testing limitations for the 100 GHz signal, we scaled the 10 GHz signal obtained by OFD to 100 GHz for a joint comparison (red line), and it can be found that the 100 GHz signal can reach the quantum noise limit without interference. It should be noted that the slightly lower phase noise of the 10 GHz OFD signal (scaled to 100 GHz) compared to the directly measured 100 GHz signal, observed at offset frequencies between 100 Hz and 10 kHz, is attributed to a minor measurement deviation.

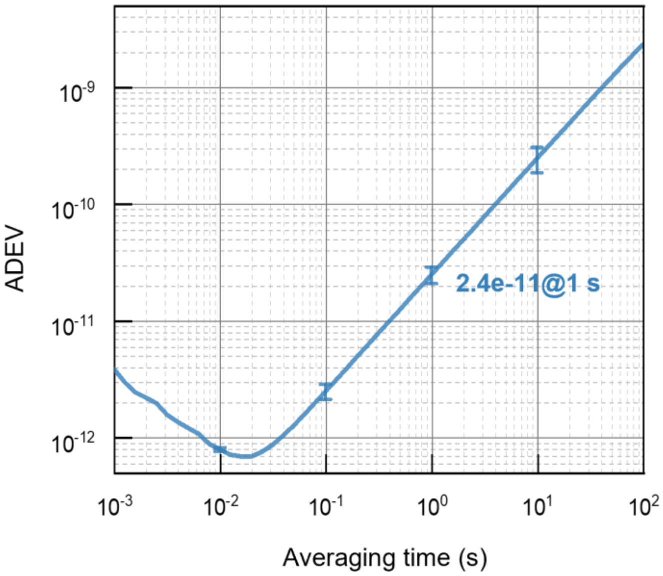

Fig. S3. Measured Allan deviation of the locked OFD. The OFD signal achieves a fractional frequency stability of 2.4×10^{-11} at 1 s averaging time.

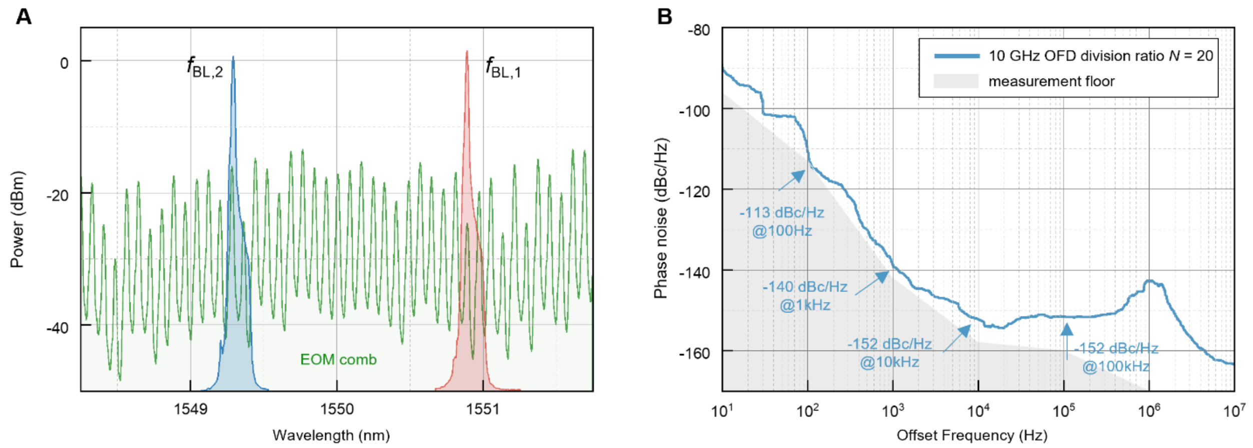

Fig. S4. Relevant demonstration of the frequency division factor *N* = 20. (**A**) Optical spectrum of the Brillouin laser references and the EOM combs divider, the 10th order modulation sidebands from both comb overlap, correspondingly N =20 and the OFD output frequency f_{OFD} is 10 GHz . (**B**) Measured phase noises of the OFD output signal after the PLL is closed. Measurement floor illustrated in shadow is derived from the product manual of RS FSWP B61.

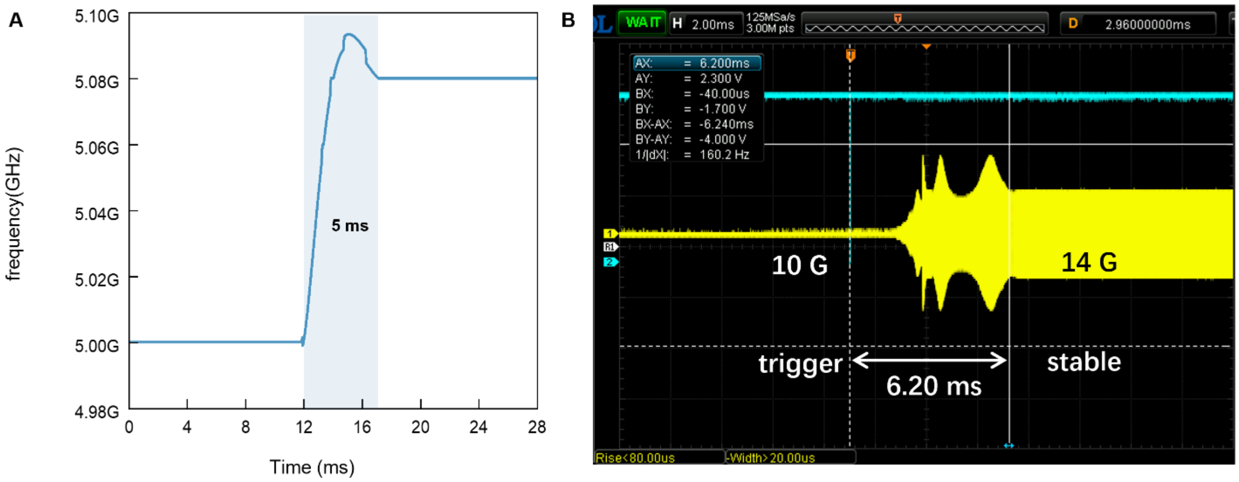

Fig. S5. Relevant frequency hopping performance measurement. (**A**) Small-step frequency hopping measurement from 5 GHz to 5.08 GHz, tested using the PN transient analysis function of the R&S FSWP phase noise analyzer. (**B**) Large-step frequency hopping measurement from 10 GHz to 14 GHz, tested using a low-speed oscilloscope with a 100 MHz bandwidth and a mixer.

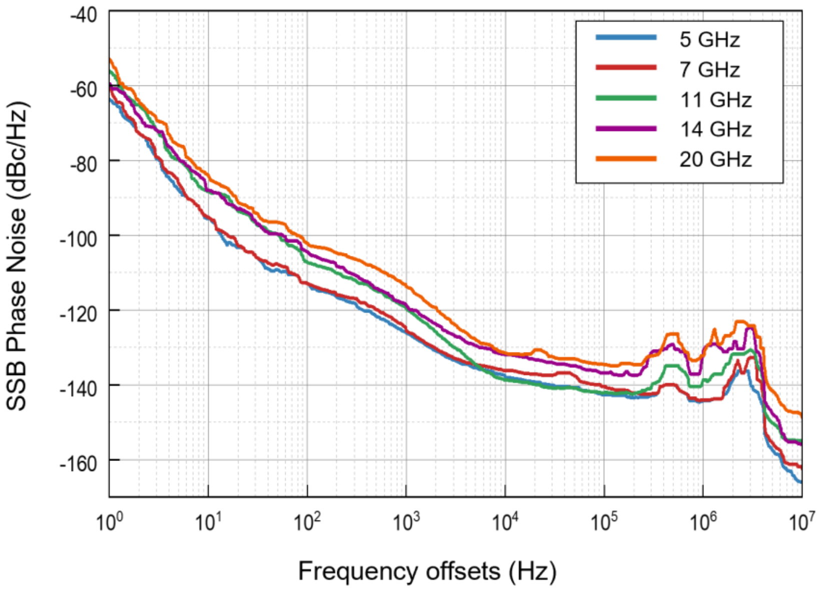

Fig. S6. Relevant phase noise performance measurement. Phase noise measurements across the full 5–20 GHz tuning range, including 5 GHz, 7 GHz, 11 GHz, 14 GHz and 20 GHz.

Tab. S1 Values of the parameters used in theoretical calculation.

Parameters	Values	Parameters	Values
Angular frequency of the CW laser (ω_c)	$2\pi \cdot 193.5 \times 10^{12}$ rad/s	Thermal conductivity (κ)	1.37 W/mK
Effective refractive index (n)	1.44	Fiber cavity length (L)	200 m
Acoustic velocity in silica fiber (V_a)	5960 m/s	Boundary condition parameter (k_{max})	3.846×10^5 m^{-1}
Planck constant (h)	6.626×10^{-34} J · s	Boundary condition parameter (k_{min})	3.848×10^4 m^{-1}
Boltzmann constant (k_B)	1.38×10^{-23} J/K	Thermal diffusivity (D)	8.2×10^{-7} m^2/s
Thermal occupation (n_{th})	568	Mechanical loss angle (ϕ_0)	0.01
Loaded Q-factor of fiber cavity (Q_T)	6.57×10^9	Cross-sectional area of the fiber (A)	19.64×10^{-8} m^2
External Q-factor of fiber cavity (Q_E)	1.64×10^{10}	Bulk modulus of silica (E_0)	1.9×10^{10} Pa
Acoustic velocity in silica fiber (V_a)	1.4 mW	Refractive index temperature coefficient (dn/dT)	9.2×10^{-6} /K
Brillouin gain linewidth (Γ)	$2\pi \cdot 30 \times 10^6$ rad/s	Temperature (T)	300 K

REFERENCES

1. C. H. Townes, A. L. Schawlow, *Microwave Spectroscopy* (Courier Corporation, 2013).

2. M. R. Khanzadi, *Phase Noise in Communication Systems—Modeling, Compensation, and Performance Analysis* (Chalmers Univ. of Technology, 2015).

3. S. A. Diddams, J. C. Bergquist, S. R. Jefferts, C. W. Oates, Standards of time and frequency at the outset of the 21st century, *Science* **306**, 1318–1324 (2004).

4. G. Serafino, F. Scotti, L. Lembo, B. Hussain, C. Porzi, A. Malacarne, S. Maresca, D. Onori, P. Ghelfi, A. Bogoni, Toward a new generation of radar systems based on microwave photonic technologies. *J. Lightwave Technol.* **37**, 643–650 (2019).

5. X. Zhang, X. Zhang, Y. Chen, W. Jin, Z. Zhou, C. Liu, C. Lao, J. Huang, J. Dong, W. Ma, W. Hu, X. Wang, J. E. Bowers, W. Li, L. Chang, Microcomb-synchronized optoelectronics. *Nat. Electron.* **8**, 322–330 (2025).

6. D. B. Leeson, A simple model of feedback oscillator noise spectrum. *Proc. IEEE* **54**, 329–330 (1966).

7. S. V. Shinde, Review of oscillator phase noise models. *Proc. Int. Multi Conf. Eng. Comput. Sci.* **2**, 733–740 (2014).

8. J. Li, X. Yi, H. Lee, S. A. Diddams, K. J. Vahala, Electro-optical frequency division and stable microwave synthesis. *Science* **345**, 309–313 (2014).

9. Y. Yao, Y. Jiang, H. Yu, Z. Bi, L. Ma, Optical frequency divider with division uncertainty at the 10^{-21} level. *Natl. Sci. Rev.* **3**, 463–469 (2016).

10. J. Li, K. Vahala, Small-sized, ultra-low phase noise photonic microwave oscillators at X-Ka bands. *Optica* **10**, 33–34 (2023).

11. T. M. Fortier, M. S. Kirchner, F. Quinlan, J. Taylor, J. Bergquist, T. Rosenband, N. Lemke, A. Ludlow, Y. Jiang, C. Oates, S. A. Diddams, Generation of ultrastable microwaves via optical frequency division. *Nat. Photon.* **5**, 425–429 (2011).

12. X. Xie, R. Bouchand, D. Nicolodi, M. Giunta, W. Hänsel, M. Lezius, A. Joshi, S. Datta, C. Alexandre, M. Lours, P.-A. Tremblin, G. Santarelli, R. Holzwarth, Y. L. Coq, Photonic microwave signals with zeptosecond-level absolute timing noise. *Nat. Photon.* **11**, 44–47 (2017).

13. W. Weng, M. H. Anderson, A. Siddharth, J. He, A. S. Raja, T. J. Kippenberg, Coherent terahertz-to-microwave link using electro-optic-modulated Turing rolls. *Phys. Rev. A* **104**, 023511 (2021).

14. E. Benkler, B. Lipphardt, T. Puppe, R. Wilk, F. Rohde, U. Sterr, End-to-end topology for fiber comb based optical frequency transfer at the 10^{-21} level. *Opt. Express* **27**, 36886–36902 (2019).

15. D. Nicolodi, B. Argence, W. Zhang, R. L. Targat, G. Santarelli, Y. L. Coq, Spectral purity transfer between optical wavelengths at the 10^{-18} level. *Nat. Photon.* **8**, 219–223 (2014).

16. K. Beha, D. C. Cole, P. Del'Haye, A. Coillet, S. A. Diddams, S. B. Papp, Electronic synthesis of light, *Optica* **4**, 406–411 (2017).

17. D. R. Carlson, D. D. Hickstein, W. Zhang, A. J. Metcalf, F. Quinlan, S. A. Diddams, S. B. Papp, Ultrafast electro-optic light with subcycle control. *Science* **361**, 1358–1363 (2018).

18. D. T. Spencer, T. Drake, T. C. Briles, J. Stone, L. C. Sinclair, C. Fredrick, Q. Li, D. Westly, B. R. Ilic, A. Bluestone, N. Volet, T. Komljenovic, L Chang, S. H. Lee, D. Y. Oh, M. Suh, K. Y. Yang, M. H. P. Pfeiffer, T. J. Kippenberg, E. Norberg, L. Theogarajan, K. Vahala, N. R. Newbury, K. Srinivasan, J. E. Bowers, S. A. Diddams, S. B. Papp, An optical-frequency synthesizer using integrated photonics. *Nature* **557**, 81–85 (2018).

19. T. C. Briles, J. R. Stone, T. E. Drake, D. T. Spencer, C. Fredrick, Q. Li, D. Westly, B. R. Ilic, K. Srinivasan, S. A. Diddams, S. B. Papp, Interlocking Kerr-microresonator frequency combs for microwave to optical synthesis, *Opt. Lett.* **43**, 2933–2936 (2018).

20. T. E. Drake, T. C. Briles, J. R. Stone, D. T. Spencer, D. R. Carlson, D. D. Hickstein, Q. Li, D. Westly, K. Srinivasan, S. A. Diddams, S. B. Papp, Terahertz-rate Kerr-microresonator optical clockwork. *Phys. Rev.* **9**, 031023 (2019).

21. T. E. Drake, J. R. Stone, T. C. Briles, S. B. Papp, Thermal decoherence and laser cooling of Kerr microresonator solitons. *Nat. Photon.* **14**, 480–485 (2020).

22. X. Xie, R. Bouchand, D. Nicolodi, M. Lours, C. Alexandre, Y. L. Coq, Phase noise characterization of sub-hertz linewidth lasers via digital cross correlation. *Opt. Lett.* **42**, 1217–1220 (2017).

23. I. Kudelin, W. Groman, Q.-X. Ji, J. Guo, M. L. Kelleher, D. Lee, T. Nakamura, C. A. McLemore, P. Shirmohammadi, S. Hanifi, H. Cheng, N. Jin, L. Wu, S. Halladay, Y. Luo, Z. Dai, W. Jin, J. Bai, Y. Liu, W. Zhang, C. Xiang, L. Chang, V. Iltchenko, O. Miller, A. Matsko, S. M. Bowers, P. T. Rakich, J. C. Campbell, J. E. Bowers, K. J. Vahala, F. Quinlan, S. A. Diddams, Photonic chip-based low-noise microwave oscillator. *Nature* **627**, 534–539 (2024).

24. I. Kudelin, P. Shirmohammadi, W. Groman, S. Hanifi, M. L. Kelleher, D. Lee, T. Nakamura, C. A. McLemore, A. Lind, D. Meyer, J. Bai, J. C. Campbell, S. M. Bowers, F. Quinlan, S. A. Diddams, An optoelectronic microwave synthesizer with frequency tunability and low phase noise. *Nat. Electron.* **7**, 1170–1175 (2024).

25. Y. He, L. Cheng, H. Wang, Y. Zhang, R. Meade, K. Vahala, M. Zhang, J. Li, Chip-scale high-performance photonic microwave oscillator. *Sci. Adv.* **10**, eado9570 (2024).

26. Q.-X. Ji, W. Zhang, P. Liu, S. Sun, W. Jin, J. Guo, J. Peters, L. Wu, A. Feshali, M. Paniccia, V. Ilchenko, J. Bowers, A. Matsko, K. Vahala, Dispersive-wave-agile optical frequency division. *Nat. Photon.* **19**, 624–629 (2025).

27. L. Cheng, M. Zhao, Y. He, Y. Zhang, R. Meade, K. Vahala, M. Zhang, J. Li, Spiral resonator referenced low noise microwave generation via integrated optical frequency division, *Photonics Res.* **13**, 1991–1996 (2025).

28. T. Tetsumoto, T. Nagatsuma, M. E. Fermann, G. Navickaite, M. Geiselmann, A. Rolland, Optically referenced 300 GHz millimetre-wave oscillator. *Nat. Photon.* **15**, 516–522 (2021).

29. S. Sun, B. Wang, K. Liu, M. W. Harrington, F. Tabatabaei, R. Liu, J. Wang, S. Hanifi, J. S. Morgan, M. Jahanbozorgi, Z. Yang, S. M. Bowers, P. A. Morton, K. D. Nelson, A. Beling, D. J. Blumenthal, X. Yi, Integrated optical frequency division for microwave and mmWave generation. *Nature* **627**, 540–545 (2024).

30. W. Loh, D. Gray, R. Irion, O. May, C. Belanger, J. Plant, P. W. Juodawlkis, S. Yegnanarayanan, Ultralow noise microwave synthesis via difference frequency division of a Brillouin resonator. *Optica* **11**, 492–497 (2024).

31. B. He, J. Yang, F. Meng, J. Yu, C. Zhang, Q.-F. Yang, Y. Zuo, Y. Lin, Z. Chen, Z. Fang, X. Xie, Highly coherent two-color laser and its application for low-noise microwave generation. *Nat Commun.* **16**, 4034 (2025).

32. S. Sun, M. W. Harrington, F. Tabatabaei, S. Hanifi, K. Liu, J. Wang, B. Wang, Z. Yang, R. Liu, J. S. Morgan, S. M. Bowers, P. A. Morton, K. D. Nelson, A. Beling, D. J. Blumenthal, X. Yi, Microcavity Kerr optical frequency division with integrated SiN photonics. *Nat. Photon.* **19**, 637–642 (2025).

33. X. Jin, Z. Xie, X. Zhang, H. Hou, B. Wu, F. Zhang, X. Zhang, L. Chang, Q. Gong, Q.-F. Yang, Microresonator-referenced soliton microcombs with zeptosecond-level timing noise. *Nat. Photon.* **19**, 630–636 (2025).

34. C. Salomon, D. Hils, J. Hall, Laser stabilization at the millihertz level. *J. Opt. Soc. Am. B* **5**, 1576–1587 (1988).

35. Q.-F. Chen, A. Nevsky, S. Schiller, Locking the frequency of lasers to an optical cavity at the 1.6×10^{-17} relative instability level. *Appl. Phy. B* **107**, 679–683 (2012).

36. L. Li, F. Liu, C. Wang, L. Chen, Measurement and control of residual amplitude modulation in optical phase modulation. *Rev. Sci. Instrum.* **83**, 043111 (2012).

37. W. Zhang, M. J. Martin, C. Benko, J. L. Hall, J. Ye, C. Hagemann, T. Legero, U. Sterr, F. Riehle, G. D. Cole, M. Aspelmeyer, Reduction of residual amplitude modulation to 1×10^{-6} for frequency modulation and laser stabilization. *Opt. Lett.* **39**, 1980–1983 (2014).

38. Y.-X. Chao, Z.-X. Hua, X.-H. Liang, Z.-P. Yue, L. You, M. Khoon Tey, Pound–Drever–Hall feedforward: Laser phase noise suppression beyond feedback. *Optica* **11**, 945–950 (2024).

39. J. Li, H. Lee, T. Chen, K. J. Vahala, Characterization of a high coherence, Brillouin microcavity laser on silicon. *Opt. Express* **20**, 20170–20180 (2012).

40. J. Hu, S. Xue, Y. Xiao, C. Wu, H. Wen, H. Zhou, Synergizing the low-phase noise characteristics of a dielectric resonator oscillator and a bichromatic Brillouin laser oscillator. *Opt. Lett.* **50**, 988–991 (2025).

41. S. Liu, Y. Yang, Y. Geng, H. Wen, H. Zhou, Fast and accurate extraction of ultrahigh quality factors from cavity ringdown measurement. *Opt. Lett.* **49**, 5399–5402 (2024).

42. M.-G. Suh, Q.-F. Yang, K. J. Vahala, Phonon-limited-linewidth of Brillouin lasers at cryogenic temperatures. *Phys. Rev. Lett.* **119**, 143901 (2017).

43. S. Gundavarapu, G. M. Brodnik, M. Puckett, T. Huffman, D. Bose, R. Behunin, J. Wu, T. Qiu, C. Pinho, N. Chauhan, J. Nohava, P. T. Rakich, K. D. Nelson, M. Salit, D. J. Blumentha, Sub-hertz fundamental linewidth photonic integrated Brillouin laser. *Nat. Photon.* **13**, 60–67 (2019).

44. L. Duan, General treatment of the thermal noises in optical fibers. *Phys. Rev. A* **86**, 023817 (2012).

45. K. H. Wanser, Fundamental phase noise limit in optical fibres due to temperature fluctuations. *Electron. Lett.* **28**, 53–54 (1992).

46. W. Loh, D. Gray, R. Maxson, D. Kharas, J. Plant, P. W. Juodawlkis, C. Sorace-Agaskar, S. Yegnanarayanan, Magic cancellation point for vibration resilient ultrastable microwave signal synthesis. arXiv:2502.08780 (2025).

47. A. Debut, S. Randoux, J. Zemmouri, Linewidth narrowing in Brillouin lasers: Theoretical analysis. *Phys. Rev. A* **62**, 023803 (2000).

48. N. T. Otterstrom, R. O. Behunin, E. A. Kittlaus, P. T. Rakich, A silicon Brillouin laser. *Science* **360**, 1113–1116 (2018).

49. Y. Qi, X. Jia, J. Wang, W. Yang, Y. Miao, X. Cai, G. Wu, Y. Li, 1.79-GHz acquisition rate absolute distance measurement with lithium niobate electro-optic comb. *Nat. Commun.* **16**, 2889 (2025).

50. D. Chelladurai, *M. Kohli*, J. Winiger, D. Moor, A. Messner, Y. Fedoryshyn, M. Eleraky, Y. Liu, H. Wang, J. Leuthold, Barium titanate and lithium niobate permittivity and Pockels coefficients from megahertz to sub-terahertz frequencies. *Nat. Mater.* **24**, 868–875(2025).

51. M. Yu, D. Barton III, R. Cheng, C. Reimer, P. Kharel, L. He, L. Shao, D. Zhu, Y. Hu, H. R. Grant, L. Johansson, Y. Okawachi, A. L. Gaeta, M. Zhang, M. Lončar, Integrated femtosecond pulse generator on thin-film lithium niobate. *Nature* **612**, 252–258 (2022).

52. J. Yang, K. V. Gasse, D. M. Lukin, M. A. Guidry, G. H. Ahn, A. D. White, J. Vučković, Titanium:sapphire-on-insulator integrated lasers and amplifiers. *Nature* **630**, 853–859 (2024).

53. P. Zhao, V. Shekhawat, M. Girardi, Z. He, V. Torres-Company, P. A. Andrekson, Ultra-broadband optical amplification using nonlinear integrated waveguides. *Nature* **640**, 918–923 (2025).

54. W. Groman, I. Kudelin, A. Lind, D. Lee, T. Nakamura, Y. Liu, M. L. Kelleher, C. A. McLemore, J. Guo, L. Wu, W. Jin, J. E. Bowers, F. Quinlan, S. A. Diddams, Photonic millimeter-wave generation beyond the cavity thermal limit. *Optica* **11**, 1583–1587 (2024).

55. W. Loh, S. Yegnanarayanan, F. O'Donnell, P. W. Juodawlkis, Ultra-narrow linewidth Brillouin laser with nanokelvin temperature self-referencing. *Optica* **6**, 152–159 (2019).

56. J. Dong, J. Huang, T. Li, L. Liu, Observation of fundamental thermal noise in optical fibers down to infrasonic frequencies. *Appl. Phys. Lett.* **108**, 021108(2016).

57. J. H. Dallyn, K. Liu, M. W. Harrington, G. M. Brodnik, P. T. Rakich, D. J. Blumenthal, R. O. Behunin, Thermal and driven noise in Brillouin lasers. *Phys. Rev. A* **105**, 043506 (2022).

58. R. O. Behunin, N. T. Otterstrom, P. T. Rakich, S. Gundavarapu, D. J. Blumenthal, Fundamental noise dynamics in cascaded-order Brillouin lasers. *Phys. Rev. A* **98**, 023832 (2018).

59. J. Li, H. Lee, K. J. Vahala, Microwave synthesizer using an on-chip Brillouin oscillator. *Nat. Commun.* **4**, 2097 (2013).
